# Editorial: Minimally invasive pediatric surgery: how to improve and overcome limitations

**DOI:** 10.3389/fsurg.2024.1446901

**Published:** 2024-07-17

**Authors:** Edoardo Bindi, Gabriele Lisi, Giuseppe Lauriti, Giovanni Cobellis

**Affiliations:** ^1^ Department of Pediatric Surgery, Azienda Ospedaliero Universitaria Ospedali Riuniti, Ancona, Italy; ^2^ Department of Clinical Specialty Science and Dentistry, Marche Polytechnic University, Ancona, Italy; ^3^ Department of Medicine and Science of Aging, University of Studies G. D'Annunzio Chieti and Pescara, Chieti, Italy

**Keywords:** minimally invasive surgery, robotic surgery, laparoscopy, thoracoscopy, pediatric surgery

**Editorial on the Research Topic** Minimally invasive pediatric surgery: how to improve and overcome limitations

The evolution of minimally invasive pediatric surgery is one of modern medicine's most exciting and progressive chapters. This practice, which originated in the development of laparoscopic surgery in adults in the 1980s, has gained a prominent position in the care of pediatric patients because of its many advantages over more invasive traditional surgical approaches. Minimally invasive pediatric surgery began to take shape in the 1990s, with the adoption of laparoscopic techniques for simple surgeries such as appendectomy and cholecystectomy. An important milestone was set in 1995 when van der Zee performed the first laparoscopic CDH repair (1). Since then, it has seen a wide expansion in techniques and applications, ranging from gastrointestinal to urological and thoracic surgeries. After that, surgical techniques have undergone continuous improvements, up to unimaginable goals, such as performing robotic-assisted surgical procedures on patients under the age of one. Technological advances have played a crucial role in the evolution of minimally invasive pediatric surgery (2). Miniaturization of surgical instruments and improvements in imaging technologies have made operating safe even in the smallest patients possible. Robotics and augmented reality systems are now frequently integrated to improve the precision and effectiveness of surgery. Robot-assisted surgery, in particular, has enabled a further decrease in incision size and an increase in precision, thanks to robotic arms that eliminate the natural tremor of the human hand and allow extremely controlled and delicate movements. The evolution of techniques and knowledge has made it possible to overcome barriers, operating with minimally invasive techniques on small patients with complex pathologies (3, 4). The clinical results of pediatric minimally invasive surgery have been extraordinary. Several studies have shown that children undergoing minimally invasive procedures experience less postoperative pain, have lower risks of infection, and enjoy faster convalescence than those treated with traditional techniques (5, 6). In addition, the reduced visual impact of scars contributes positively to young patients' psychological acceptance of surgical treatment.

The goal of this special issue was to collect publications that could best describe and summarize not only the evolution of minimally invasive pediatric surgery but also set goals for future developments.

Robot-assisted surgery has made it easier for pediatric surgeons to perform essential reconstructive-type procedures. One of the most significant examples is ureterovesical junction surgery, in which the robotic approach demonstrates its advantages. In their work, He et al. described how the technique of modified Lich-Gregoir direct nipple ureteral extravesical reimplantation can help maintain the physiological direction of the ureter and at the same time enhance the effectiveness of antireflux in robotic surgery. In addition, design of a single-port-plus-one wound can produce a cosmetic appearance by concentrating and hiding the wound around the umbilicus. This modified reimplantation procedure has the potential to become a promising technique in the robot-assisted treatment of primary obstructive megaureter.

Robot-assisted surgery is also catching on in the field of oncology surgery, in selected cases, showing the same good results as in other areas. Liang et al. described a case of a 3-year-old patient with a periampullary rhabdomyosarcoma who underwent a robotic pylorus-preserving pancreatoduodenectomy. With the assistance of a modern robotic system, they performed an R0 resection and a reconstruction with end-to-end pancreatojejunostomy, end-to-side hepaticojejunostomy, and duodenojejunostomy, without fatal complications such as pancreatic fistula or leak. The case reported demonstrates that also this kind of procedure in pediatric patients is safe and effective without intra- or postoperative complications.

An important part of minimally invasive surgery remains the domain of techniques such as laparoscopy and thoracoscopy. In this special issue, we have collected interesting experiences from this point of view. Laparoscopic herniorrhaphy in pediatric patients has become a routine procedure, often performed in day surgery. Zhang et al. reported a large series of 848 patients undergoing single-port laparoscopic herniorrhaphy. They described satisfactory results with no cases of conversion and 2 patients presenting with recurrence. They stated that this intervention presents numerous benefits, including the utilization of uncomplicated instruments, straightforward operation, a clear curative impact, minimal tissue damage, rapid recovery, and the absence of scarring. Another interesting example is that of Jung, who reports the case of a newborn presented with a rare combination of esophageal atresia with tracheoesophageal fistula and duodenal atresia, which was successfully managed using minimally invasive surgical techniques. The neonate underwent a thoracoscopic ligation of the tracheoesophageal fistula (TEF) and a laparoscopic duodeno-duodenostomy on the same day, resulting in stabilized vital signs. Ten days after the initial operation, a thoracoscopic esophago-esophagostomy was successfully performed. This report details a recent successful experience with a two-stage operation conducted without gastrostomy and utilizing minimally invasive surgical techniques. This approach underscores the evolving potential for neonatal treatment strategies in managing such complex cases.

Talking about minimally invasive surgery also means addressing how to enable surgeons, starting with residents, to acquire a progressive learning curve to make them autonomous even in the most complex cases. In this sense, the development of training programs involving faithful anatomical models becomes essential to ensure the development of the basic skills needed to approach this type of procedure. In this special issue, we have collected two original types of research dealing with different aspects of the same topic. Zahradnikova et al. created a 3D printed model for thoracoscopic repair of esophageal atresia with tracheoesophageal fistula and created a program in which 18 participants with different surgical experiences practiced. The results emphasized that this type of model proved useful as a training tool, partly because of its realism. The authors stated that due to its reusability, and suitability for individual participants, this model holds promise as a training tool for thoracoscopic procedures among surgeons. Miyano et al., on the other hand, described a cadaver teaching program to evaluate the usefulness of remote teaching. The results of their study show, that although inferior to hands-on practice, remote observation of minimally invasive procedures provides good results and can be particularly useful for training programs in resource-limited settings.

Lastly, when talking about pediatric minimally invasive surgery, and addressing the topic of what its developments might be, the focus on patients cannot be lost sight of. Pediatric patients require different management and attention than adults, and to neglect this is to disregard the overall health of patients. From this point of view, one of the main aspects is the management of the patient's preoperative anxiety. Franconi et al., designed a study, using a humanoid robot that would interact with patients, accompanying them as they entered the operating room. The purpose of this study was to determine whether the use of a humanoid robot could reduce preoperative anxiety levels in children. This work had the interesting result of showing that a non-pharmacological intervention like a humanoid robot reduces anxiety in children during the pre-operative time and it might be an attractive solution to optimize perioperative care in children.

In conclusion, we can say that the success of this Special Issue, in addition to the important number of articles collected, also lies in the fact that it has shown how minimally invasive surgery, even in pediatric settings, is a field where important milestones have been achieved, without ceasing to look toward future goals of further development.

## References

[1] van der Zee DC Bax NM. Laparoscopic repair of congenital diaphragmatic hernia in a 6-month-old child. Surg Endosc. (1995) 9(9):1001–3. 10.1007/BF00188460 7482203

[2] Esposito C Masieri L Castagnetti M Pelizzo G De Gennaro M Lisi G Current status of pediatric robot-assisted surgery in Italy: epidemiologic national survey and future directions. J Laparoendosc Adv Surg Tech A. (2023) 33(6):610–4. 10.1089/lap.2019.0516 31916914

[3] Bindi E Todesco C Nino F Torino G Gentilucci G Cobellis G. Robotic surgery: is there a possibility of increasing its application in pediatric settings? A single-center experience. Children (Basel). (2022) 9(7):1021. 10.3390/children9071021 35884005 PMC9325175

[4] Cobellis G Bindi E. Pyeloplasty in children with ureteropelvic junction obstruction and associated kidney anomalies: can a robotic approach make surgery easier? Children (Basel). (2023) 10(9):1448. 10.3390/children10091448 37761409 PMC10527626

[5] Bindi E Nino F Bollettini T Chiarella E Cobellis G. Robot-assisted excision of partially obstructing ureteral fibroepithelial polyp in a child: a case report and review of the literature. J Pediatr Surg Case Rep. (2022) 84:102393. 10.1016/j.epsc.2022.102393

[6] Pierangeli F Bindi E Cruccetti A Nino F Gentilucci G Cobellis G. Robotic-assisted excision of a giant ureteral stump in a child: case report and non-systematic review of the literature. J Pediatr Surg Case Rep. (2023) 93:102623. 10.1016/j.epsc.2023.102623

